# Temporal analysis of gestational and congenital syphilis indicators in Brazil: toward the elimination of vertical transmission by 2030?

**DOI:** 10.1590/1980-549720250028

**Published:** 2025-06-02

**Authors:** Gabriel Pavinati, Lucas Vinícius de Lima, Marjorie Fairuzy Stolarz, Melissa Ferrari Gomes, Sidnei Nathan Soares Turquino, Gabriela Tavares Magnabosco

**Affiliations:** I Universidade Estadual de Maringá, Graduate Program in Nursing – Maringá (PR), Brazil.

**Keywords:** Syphilis, Syphilis, congenital, Infectious disease transmission, vertical, Sustainable development, Time series studies

## Abstract

**Objective:**

To analyze, at national and regional levels, the trend of proxy indicators for the progress toward the elimination of the vertical transmission of syphilis in Brazil.

**Methods:**

This is an ecological study that assessed the annual percent change (APC) of indicators related to gestational and congenital syphilis.

**Results:**

An APC of 11.15% was observed in the detection rate of gestational syphilis (95% confidence interval — 95%CI 2.78–23.19), with stability in congenital syphilis across all regions. There was a reduction of prenatal care coverage among pregnant women with syphilis (APC=-7.34%; 95%CI -10.15; -5.33), and also a decrease in late diagnosis of the infection (APC=-10.77%; 95%CI -14.29; -8.79).

**Conclusions:**

We evidenced challenges in achieving the 2030 elimination targets, highlighting the need for adjustments in current public policies on syphilis prevention and control.

## INTRODUCTION

Congenital syphilis remains a challenge for health public policies in Brazil. Despite effective treatments, the infection persists because of failures in prenatal care adherence and treatment of infected pregnant women^1^. Researchers show an increase in gestational and congenital syphilis rates in the country, showing problems in early screening and adherence to care during pregnancy^1,2^.

The elimination of vertical transmission of the infection by 2030 is in the list of the Sustainable Development Goals and the *Programa Brasil Saudável* [Healthy Brazil Program]^3,4^. In Brazil, the control of congenital syphilis depends on the quality of primary health care, especially in prenatal care. Insufficient coverage and latent inequalities in access to services in the territory are obstacles to achieving this goal^1,2^.

Thus, knowing temporal trends of infection indicators is paramount for (re)directing maternal and child health policies and programs, so that actions are sustainable and effective more forcefully^2,3^. Therefore, our objective was to analyze the temporal trend of operational indicators related to the elimination of the vertical transmission of syphilis in Brazil.

## METHODS

This is a time series study of operational indicators of syphilis in Brazil and its regions with data from the Notifiable Diseases Information System (*Sistema de Informação de Agravos de Notificação*), the Live Birth Information System (*Sistema de Informação sobre Nascidos Vivos)*, and the Brazilian Institute of Geography and Statistics. Based on the time frame of a decade (2014–2023), four indicators were created related to the control of the vertical transmission of syphilis:

- Detection rate of syphilis in pregnant women: number of syphilis cases detected in pregnant women, divided by the total number of live births (LB) of mothers living in the same year and place, and the result multiplied by one thousand;
- Percentage of pregnant women with syphilis who received prenatal care: number of pregnant women with syphilis who received prenatal care, divided by the number of pregnant women with syphilis, in the same year and place, and the result multiplied by one hundred;
- Percentage of pregnant women with late diagnosis of syphilis: number of pregnant women diagnosed in or after childbirth, divided by the number of pregnant women with syphilis, in the same year and place, and the result multiplied by one hundred;
- Incidence rate of congenital syphilis: number of new confirmed cases of congenital syphilis, divided by the total number of LB of mothers living in the same year and place, and the result multiplied by one thousand.

Trend analysis was carried out with joinpoint regression models. The evolution of the historical series was analyzed for each indicator (dependent variable) in Brazil and in the five regions (North, Northeast, Southeast, South, and Midwest), depending on the years (independent variable). The indicators were logarithmized and the models were adjusted to the first order autocorrelation, when present.

The annual percent change (APC) and their 95% confidence intervals (95%CI) illustrated the trends: Positive and negative APC indicated increasing and decreasing trends, respectively, when 95%CI values were different from 0. The Joinpoint Regression Program was used. Based on Resolution No. 674/2024 of the National Health Council, ethical assessment was waived.

## RESULTS

**Figure 1:**
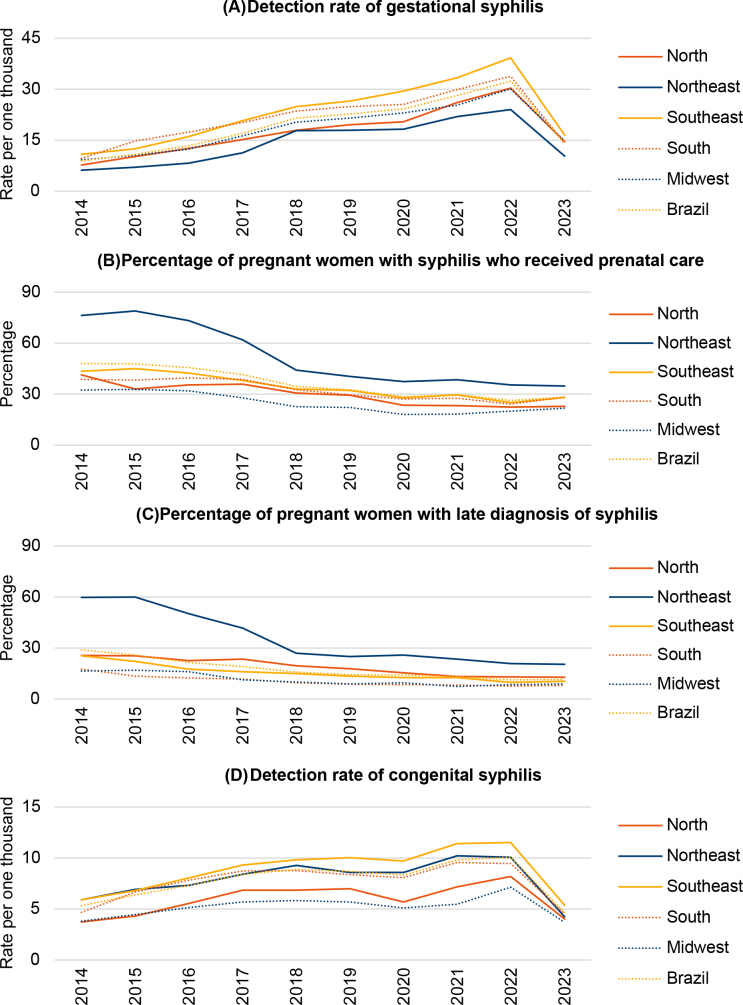
Historical series of epidemiological and operational indicators of cases of gestational and congenital syphilis, Brazil, 2014–2023.

In all regions of Brazil, the historical series of the indicators points out that, after escalating the number of cases, there was a reduction in the detection of gestational and congenital syphilis as of 2022. In addition, since 2018, a plateau was observed in the percentages of pregnant women with syphilis who received prenatal care and who had a late diagnosis for the disease, with a small variation in the period (Figure 1).

We identified, in all regions, increases in the detection rates of gestational syphilis, especially in the North and Northeast, whereas the congenital form was stable. These trends were followed by a reduction in the percentages of pregnant women who received prenatal care and who had late diagnosis, with APC of -7.34% (95%CI -10.15; -5.33) and -10.77% (95%CI -14.29; -8.79), respectively (Table 1).

**Table 1:** Temporal trends of epidemiological and operational indicators of cases of gestational and congenital syphilis, Brazil, 2014–2023.

Location	APC (95%CI)	Trend
**Detection rate of syphilis in pregnant women**		
North region	12.26 (5.81–21.09)	Increasing
Northeast region	12.26 (1.10–30.62)	Increasing
Southeast region	11.45 (2.94–23.05)	Increasing
South region	8.79 (1.27–18.15)	Increasing
Midwest region	10.52 (2.87–21.36)	Increasing
Brazil	11.15 (2.78–23.19)	Increasing
**Percentage of pregnant women with syphilis who received prenatal care**		
North region	-6.69 (-9.04; -4.95)	Decreasing
Northeast region	-9.39 (-16.33; -5.52)	Decreasing
Southeast region	-6.82 (-8.90; -4.68)	Decreasing
South region	-5.22 (-8.19; -2.95)	Decreasing
Midwest region	-5.06 (-11.94; -0.50)	Decreasing
Brazil	-7.34 (-10.15; -5.33)	Decreasing
**Percentage of pregnant women with late diagnosis of syphilis**		
North region	-8.35 (-11.11; -6.39)	Decreasing
Northeast region	-12.84 (-20.13; -8.36)	Decreasing
Southeast region	-9.08 (-11.99; -6.49)	Decreasing
South region	-9.10 (-12.53; -6.32)	Decreasing
Midwest region	-8.39 (-15.08; -4.33)	Decreasing
Brazil	-10.77 (-14.29; -8.79)	Decreasing
**Incidence rate of congenital syphilis**		
North region	4.30 (-3.51–13.59)	Stable
Northeast region	3.17 (-3.07–10.06)	Stable
Southeast region	3.86 (-3.00–11.71)	Stable
South region	2.53 (-4.05–9.86)	Stable
Midwest region	2.79 (-1.04–6.73)	Stable
Brazil	3.29 (-2.93–10.25)	Stable

APC: Annual Percent Change; 95%CI: 95% confidence interval.

## DISCUSSION

Brazil and its regions presented homogeneous trends for the operational indicators. There was an increase in the detection rates of gestational syphilis and stability in the detection of congenital syphilis. Furthermore, we verified a decreasing trend of pregnant women with the infection who received prenatal care, although the indicator regarding late diagnosis has improved, with decrease in all locations.

The elimination of vertical transmission of syphilis in the country is an ambitious goal, and the epidemiological scenario presented evidences how far the country and its regions are from reaching it. The reduction in the number of cases in pregnant women and the early diagnosis and timely treatment, based on engaging the pregnant woman and the partner in prenatal care, are necessary efforts to achieve this goal.

Despite the fact that we showed stability in congenital syphilis rates, authors of studies on Brazilian states showed a significant increase, such as in Paraná^1^, Minas Gerais^5^, and Amazonas^6^. This points to the need for investigations on the regional particularities in access and adherence to prenatal care in different locations, given the possible inequalities in the coverage of these services.

Fluctuations in the incidence rates of syphilis in the years corresponding to the COVID-19 pandemic may result from underreporting and underdetection of cases. Moreover, the underfunding of primary health care due to the extinction of the fixed and variable floor from 2019 to 2024 impacted the actions of primary health care, especially those developed by family health teams.

We emphasize the need for structuring and permanent education of healthcare teams and intersectoral partners, especially social assistance, for comprehensive care. Accordingly, it is worth highlighting the contribution potential of the *Programa Brasil Saudável*, which can favor the articulation between sectors for holistic assistance to the population.

We underline the need for strengthening primary health care as a priority point of the healthcare network. A better allocation of resources for resuming the role of family health in prenatal activities can represent the basis for coping with syphilis. Only through integrated and proactive actions will it be possible to achieve the goals set for 2030.

Despite the limitations inherent in the use of secondary data, which may not reliably represent the epidemiology of syphilis, we believe that the results of the present research serve as an alert to health authorities and can justify the redirection of actions to tackle the infection, especially after the dismantling of the healthcare network caused by the COVID-19 pandemic.

## Data Availability

All datasets were generated or analyzed in the current study.
