# Supplementary material for: Temporal analysis of gestational and congenital syphilis indicators in Brazil: toward the elimination of vertical transmission by 2030?
Source: Rev Bras Epidemiol. 2025 Jun 2;28:e250028. doi: 10.1590/1980-549720250028 (PMC12129237; doi:10.1590/1980-549720250028)
Supplement: Translation

# Translation: Análise temporal dos indicadores da sífilis gestacional e congênita no Brasil: rumo à eliminação da transmissão vertical até 2030?

**DOI:** 10.1590/1980-549720250028.2

**Authors:** Gabriel Pavinati, Lucas Vinícius de Lima, Marjorie Fairuzy Stolarz, Melissa Ferrari Gomes, Sidnei Nathan Soares Turquino, Gabriela Tavares Magnabosco

**Affiliations:** I Universidade Estadual de Maringá, Graduate Program in Nursing – Maringá (PR), Brasil.

**Keywords:** Sífilis, Sífilis congênita, Transmissão vertical de doenças infecciosas, Desenvolvimento sustentável, Estudos de séries temporais

## Abstract

**Objetivo:**

Analisar, em níveis nacional e regional, a tendência de indicadores *proxies* do progresso para eliminação da transmissão vertical da sífilis no Brasil.

**Métodos:**

Estudo ecológico que avaliou a variação percentual anual (VPA) de indicadores relacionados à sífilis gestacional e congênita.

**Resultados:**

Observou-se VPA de 11,15% na taxa de detecção de sífilis gestacional (intervalo de confiança de 95% – IC95% 2,78–23,19), com estabilidade na sífilis congênita em todas as regiões. A cobertura de pré-natal entre gestantes com sífilis apresentou redução (VPA=-7,34%; IC95% -10,15; -5,33), e o diagnóstico tardio da infecção também decresceu (VPA=-10,77%; IC95% -14,29; -8,79).

**Conclusões:**

Os achados evidenciam dificuldades no alcance das metas de eliminação até 2030, indicando a necessidade de ajustes nas políticas públicas de prevenção e controle da sífilis.

## INTRODUÇÃO

A sífilis congênita permanece como um desafio para as políticas públicas de saúde no Brasil. A despeito de tratamentos eficazes, a infecção persiste por causa das falhas no acompanhamento pré-natal e no tratamento de gestantes infectadas^1^. Estudos mostram aumento nas taxas de sífilis gestacional e congênita no país, evidenciando problemas na triagem precoce e na adesão ao cuidado durante a gravidez^1,2^.

A eliminação da transmissão vertical da infecção até 2030 está no rol dos Objetivos do Desenvolvimento Sustentável e do Programa Brasil Saudável^3,4^. No Brasil, o controle da sífilis congênita depende da qualidade da atenção primária à saúde, sobretudo no pré-natal. A cobertura insuficiente e as desigualdades latentes no acesso aos serviços no território são obstáculos ao alcance dessa meta^1,2^.

Assim, conhecer tendências temporais dos indicadores da infecção é fundamental no (re)direcionamento das políticas e dos programas de saúde materno-infantil, de modo que as ações sejam sustentáveis e efetivas de forma mais contundente^2,3^. Portanto, objetivou-se analisar a tendência temporal de indicadores operacionais relacionados à eliminação da transmissão vertical da sífilis no Brasil.

## MÉTODOS

Estudo de séries temporais de indicadores operacionais da sífilis no Brasil e suas regiões com dados do Sistema de Informação de Agravos de Notificação, do Sistema de Informação sobre Nascidos Vivos e do Instituto Brasileiro de Geografia e Estatística. Com base no recorte temporal de uma década (2014–2023), foram produzidos quatro indicadores que tangenciam o controle da transmissão vertical da sífilis:

- Taxa de detecção de sífilis em gestantes: número de casos de sífilis detectados em gestantes, dividido pelo número total de nascidos vivos (NV) de mães residentes no mesmo ano e local, e o resultado multiplicado por 1.000;
- Percentual de gestantes com sífilis que realizaram o pré-natal: número de gestantes com sífilis que realizaram pré-natal, dividido pelo número de gestantes com sífilis, no mesmo ano e local, e o resultado multiplicado por 100;
- Percentual de gestantes com diagnóstico tardio de sífilis: número de gestantes com diagnóstico no parto ou depois dele, dividido pelo número de gestantes com sífilis, no mesmo ano e local, e o resultado multiplicado por 100;
- Taxa de incidência de sífilis congênita: número de casos novos confirmados de sífilis congênita, dividido pelo número total de NV de mães residentes no mesmo ano e local, e o resultado multiplicado por 1.000.

Empregou-se a análise de tendência com modelos de regressão *joinpoint*. A evolução da série histórica foi analisada para cada indicador (variável dependente) no Brasil e nas cinco regiões (Norte, Nordeste, Sudeste, Sul e Centro-Oeste), em função dos anos (variável independente). Os indicadores foram logaritmizados e os modelos ajustados à autocorrelação de primeira ordem, quando presente.

As variações percentuais anuais (VPA) e seus intervalos de confiança de 95% (IC95%) ilustraram as tendências: VPA positivas e negativas indicaram tendências crescentes e decrescentes, respectivamente, quando os IC95% foram diferentes de 0. Usou-se o Joinpoint Regression Program. Com base na Resolução nº 674/2024 do Conselho Nacional de Saúde, dispensou-se a apreciação ética.

## RESULTADOS

Em todas as regiões do Brasil, a série histórica dos indicadores aponta que, após escalada no número de casos, houve redução na detecção de sífilis gestacional e congênita a partir de 2022. Além disso, desde 2018, observa-se um platô nos percentuais de gestantes com sífilis que realizaram o pré-natal e que tiveram o diagnóstico tardio para doença, com pequena variação no período (Figura 1).

**Figura 1:** Séries históricas dos indicadores epidemiológicos e operacionais dos casos de sífilis gestacional e congênita, Brasil, 2014–2023.

Todas as regiões apresentaram incrementos nas taxas de detecção da sífilis gestacional, especialmente no norte e nordeste, ao passo que a forma congênita foi estável. Essas tendências foram acompanhadas pela redução nos percentuais de gestantes que realizaram o pré-natal e que tiveram o diagnóstico tardio, com VPA de -7,34% (IC95% -10,15; -5,33) e -10,77% (IC95% -14,29; -8,79), respectivamente (Tabela 1).

**Tabela 1:** Tendências temporais dos indicadores epidemiológicos e operacionais dos casos de sífilis gestacional e congênita, Brasil, 2014–2023.

Local	VPA (IC95%)	Tendência
**Taxa de detecção de sífilis em gestantes**		
Região Norte	12,26 (5,81–21,09)	Aumento
Região Nordeste	12,26 (1,10–30,62)	Aumento
Região Sudeste	11,45 (2,94–23,05)	Aumento
Região Sul	8,79 (1,27–18,15)	Aumento
Região Centro-Oeste	10,52 (2,87–21,36)	Aumento
Brasil	11,15 (2,78–23,19)	Aumento
**Percentual de gestantes com sífilis que realizaram pré-natal**		
Região Norte	-6,69 (-9,04; -4,95)	Redução
Região Nordeste	-9,39 (-16,33; -5,52)	Redução
Região Sudeste	-6,82 (-8,90; -4,68)	Redução
Região Sul	-5,22 (-8,19; -2,95)	Redução
Região Centro-Oeste	-5,06 (-11,94; -0,50)	Redução
Brasil	-7,34 (-10,15; -5,33)	Redução
**Percentual de gestantes com diagnóstico tardio de sífilis**		
Região Norte	-8,35 (-11,11; -6,39)	Redução
Região Nordeste	-12,84 (-20,13; -8,36)	Redução
Região Sudeste	-9,08 (-11,99; -6,49)	Redução
Região Sul	-9,10 (-12,53; -6,32)	Redução
Região Centro-Oeste	-8,39 (-15,08; -4,33)	Redução
Brasil	-10,77 (-14,29; -8,79)	Redução
**Taxa de incidência de sífilis congênita**		
Região Norte	4,30 (-3,51–13,59)	Estável
Região Nordeste	3,17 (-3,07–10,06)	Estável
Região Sudeste	3,86 (-3,00–11,71)	Estável
Região Sul	2,53 (-4,05–9,86)	Estável
Região Centro-Oeste	2,79 (-1,04–6,73)	Estável
Brasil	3,29 (-2,93–10,25)	Estável

VPA: variação percentual anual; IC95%: intervalo de confiança de 95%.

## DISCUSSÃO

O Brasil e suas regiões apresentaram tendências homogêneas para os indicadores operacionais. Evidenciaram-se aumento nas taxas de detecção de sífilis gestacional e estabilidade na detecção da sífilis congênita. Ainda, verificou-se tendência decrescente das gestantes com a infecção que realizaram o pré-natal, embora o indicador referente ao diagnóstico tardio tenha melhorado, com redução em todas as localidades.

A eliminação da transmissão vertical da sífilis no país é uma meta ambiciosa, e o cenário epidemiológico apresentado demonstra a distância que o país e suas regiões têm em alcançá-la. A redução do número de casos em gestantes e o diagnóstico precoce e tratamento oportuno, pautados em uma vinculação ao pré-natal da gestante e do parceiro, são esforços necessários para que se consiga alcançar essa meta.

A despeito de este estudo ter mostrado estabilidade nas taxas de sífilis congênita, pesquisas em estados brasileiros evidenciaram aumento significativo, como Paraná^1^, Minas Gerais^5^ e Amazonas^6^. Isso aponta para a necessidade de investigações sobre as particularidades locorregionais no acesso e na vinculação ao pré-natal em diferentes localidades, dadas as possíveis iniquidades na cobertura desses serviços.

As flutuações das taxas de incidência da sífilis nos anos correspondentes à pandemia de COVID-19 podem decorrer de subnotificação e subdetecção de casos. Ademais, o subfinanciamento da atenção primária por causa da extinção do piso fixo e variável no período de 2019 a 2024 impactou as ações da atenção primária, especialmente as desenvolvidas pelas equipes de saúde da família.

Reforça-se a necessidade de estruturação e educação permanente de equipes de profissionais de saúde e parceiros intersetoriais, sobretudo da assistência social, para o cuidado integral. Nessa lógica, cumpre destacar o potencial de contribuição do Programa Brasil Saudável, o qual tem potencial de favorecer a articulação entre setores para a assistência holística à população.

Esta comunicação sublinha a necessidade de fortalecimento da atenção primária à saúde como ponto prioritário da rede de atenção. A melhor alocação de recursos para a retomada do protagonismo da saúde da família nas atividades de pré-natal pode representar o alicerce no enfrentamento da sífilis. Somente mediante ações integradas e proativas será possível alcançar as metas estabelecidas para 2030.

Apesar das limitações inerentes ao uso dos dados secundários, que podem não representar fidedignamente a epidemiologia da sífilis, acredita-se que os resultados da presente pesquisa sirvam como alerta às autoridades sanitárias e possam fundamentar o redirecionamento das ações de enfrentamento da infecção, especialmente após a desestruturação da rede de saúde ocasionada pela pandemia de COVID-19.

## Data Availability

Todos os conjuntos de dados foram gerados ou analisados no estudo atual.
